# Real-Time Monitoring of Levetiracetam Effect on the Electrophysiology of an Heterogenous Human iPSC-Derived Neuronal Cell Culture Using Microelectrode Array Technology

**DOI:** 10.3390/bios11110450

**Published:** 2021-11-12

**Authors:** Andrea Di Credico, Giulia Gaggi, Pascal Izzicupo, Laura Ferri, Laura Bonanni, Giovanni Iannetti, Angela Di Baldassarre, Barbara Ghinassi

**Affiliations:** 1 Department of Medicine and Aging Sciences, “G. D’Annunzio” University of Chieti-Pescara, 66100 Chieti, Italy; andrea.dicredico@unich.it (A.D.C.); izzicupo@unich.it (P.I.); laura.bonanni@unich.it (L.B.); b.ghinassi@unich.it (B.G.); 2 Beth Israel Deaconess Medical Center, Harvard Medical School Initiative for RNA Medicine, Harvard Medical School, Boston, MA 02115, USA; ggaggi@bidmc.harvard.edu; 3 Department of Neuroscience, Imaging and Clinical Sciences, “G. D’Annunzio” University of Chieti-Pescara, 66100 Chieti, Italy; ferrilaura@outlook.it; 4 Faculty of Medicine and Dentistry, University of Rome La Sapienza, 00185 Rome, Italy; iannetti.1752004@studenti.uniroma1.it

**Keywords:** microelectrode array, biosensors, real-time monitoring, iPSC-derived neurons, drug screening, Levetiracetam, electrophysiology

## Abstract

Levetiracetam (LEV) is a broad-spectrum and widely used antiepileptic drug that also has neuroprotective effects in different neurological conditions. Given its complex interaction with neuronal physiology, a better comprehension of LEV effects on neurons activity is needed. Microelectrode arrays (MEAs) represent an advanced technology for the non-invasive study of electrophysiological activity of neuronal cell cultures. In this study, we exploited the Maestro Edge MEA system, a platform that allows a deep analysis of the electrical network behavior, to study the electrophysiological effect of LEV on a mixed population of human neurons (glutamatergic, GABAergic and dopaminergic neurons, and astrocytes). We found that LEV significantly affected different variables such as spiking, single-electrode bursting, and network bursting activity, with a pronounced effect after 15 min. Moreover, neuronal cell culture completely rescued its baseline activity after 24 h without LEV. In summary, MEA technology confirmed its high sensitivity in detecting drug-induced electrophysiological modifications. Moreover, our results allow one to extend the knowledge on the electrophysiological effects of LEV on the complex neuronal population that resembles the human cortex.

## 1. Introduction

Levetiracetam (LEV) is a second-generation, broad-spectrum antiepileptic, generally prescribed for the treatment of myoclonic, partial, and primary generalized tonic-clonic seizures [1,2]. Currently, little is known about the mechanisms through which LEV exerts its anti-epileptic action [3]; they seem to include the binding to the synaptic vesicle protein 2A (SV2A) with the consequent modification of glutamate and GABA release [4]; the inhibition of N-type Ca^2+^ channels and of the Ca^2+^ release from intracellular stores; the inhibition of excessive synchronized activity between neurons [5]. LEV also plays a protective role in different conditions. For example, LEV promotes neuroprotection of motor neurons in humans (h) iPSCs-Derived Spinal Muscular Atrophy Model [6] and protects rat brain cells from the excitotoxic effect of quinolinic acid [7]. Thus, more studies investigating complex electrophysiological behavior in neurons in response to LEV administration are needed to gain further insights into this drug.

Microelectrode arrays (MEAs) are non-invasive biosensors that can reliably detect the electrophysiological spontaneous activity of thousands of neurons [8]. MEAs consist of a high number of electrodes that can be of different materials (e.g., gold, poly(3,4-ethylenedioxythiophene) polystyrene sulfonate—PEDOT) [9] usually implanted at the base of tissue culture wells that allows to record (and eventually stimulate) the activity of cells capable to generate electrical signals (e.g., neurons, cardiomyocytes) [10,11,12] in a label-free manner and in real-time. In neuronal cell culture, MEAs allow one to record the Extracellular Action Potentials (EAP, also called spikes) [10,13]. In addition to the spike rate and timing, last-generation MEAs can also provide higher-level analysis, recording single-electrode bursting and network bursting activity [14]. These features provide important information regarding the electrophysiological activity and connectivity inside the neuronal cell culture [15]. In addition, MEAs can reveal EAP waveforms, a measure that reflects extracellular voltage fields produced by a neuron’s internal action potentials [16,17]. EAP waveform analysis is an interesting tool to consider since it was seen that pharmacological treatment can alter its amplitude and waveform [8,18], and different neuron types can also produce different waveforms [16].

hiPSCs-derived neurons represent a valid model for the in vitro assessment of electrophysiological behavior in response to specific drugs [19]; in particular, iPSCs-derived cortical glutamatergic neurons mimic the activity pattern of in vivo models [20,21,22] especially when co-cultured with astrocytes, which increase their maturation efficiency and enhance their spontaneous activity [10]. More recently, it was seen that co-cultures of different human central nervous system (CNS) cells (glutamatergic, GABAergic, dopaminergic neurons, and astrocytes), allow the formation of physiologically relevant neural networks and recapitulate the interplay between excitatory and inhibitory neurons, representing an optimal neurobiological model for basal electrophysiology analysis, disease models and drug screening [23]. Therefore, the use of MEAs technology on this mixed hiPSCs-derived neuronal cell population represents the gold standard method to evaluate complex electrophysiological activity in vitro.

To date, different studies investigated the effect of antiepileptic drugs using MEAs [10,24]. However, no studies have investigated the LEV effect during an extended time course on a mixed population of human neuronal cells. Thus, the present study aimed to perform a comprehensive analysis of the electrophysiological response (spiking, single-electrode bursting, and network bursting) to LEV on the neural networks, to extend the knowledge about the electrophysiological effects of this drug.

## 2. Materials and Methods

### 2.1. Cell Culture

hiPSC-derived hCNS cells (glutamatergic, GABAergic and dopaminergic neurons, and astrocytes), were obtained from Ncardia (Ncyte CNS Neuron Kit II, Ncardia). After thawing, cells were cultured in Complete Ncyte CNS Medium (Ncyte Neuronal Basal Medium completed with Neural supplement, all from Ncardia) following the manufacture’s suggestions. The mixed population consisted approximately of 30% GABAergic, 30% glutamatergic, 10% dopaminergic neurons, and 10% astrocytes. The electrical activity of the hCNS cells was monitored weekly. After about 1 month of culture, hCNS Neuron Cells showed synchronous electrical activity patterns along with regular occurrence of sharp network bursts.

### 2.2. MEA Plates Coating, and Seeding of Ncyte CNS Cells

**Figure 1:**
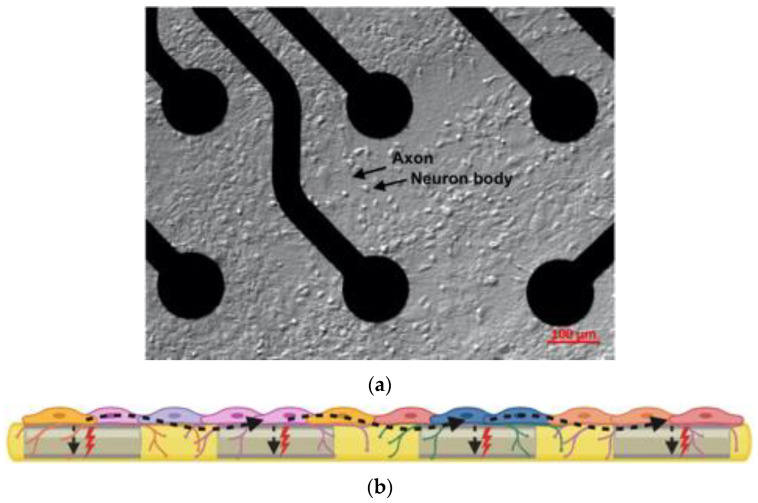
(**a**) hCNS-Neuron Cells culture on an MEA well, on the top of the recording electrodes; the black arrows highlights an axon and a cellular body, 10× magnification; (**b**) a schematic “cross-sectional” overview of the MEA plate technology: spontaneous extracellular action potentials or spikes (red thunders) originated by different neuron types are recorded by the electrodes (gray rectangles). Besides the spontaneous cellular spikes registered by a single or multiple electrodes, the electrochemical signal can also travel across the neural network, indicating synaptic communication (denoted by undulated black dashed arrows).

For the MEA study, cells were cultured on a 24-Well CytoView MEA plate (M384-tMEA-24W, Axion Biosystem, Atlanta, GA, USA). This kind of MEA plate ensures high-quality signals delivered by poly(3,4-ethylenedioxythiophene) polystyrene sulfonate (PEDOT) microelectrode. In Figure 1, both the cell culture on the recording electrodes and a schematic figure of the 24-Well CytoView MEA plate are shown. Before cell seeding, plates were precoated with CNS plate coating solution (Ncardia) and Poli-ethyleneimine 50% (*w*/*w*) (P3143, Sigma-Aldrich) at 0.1% for 30 min and then with Laminin (L2020, Sigma-Aldrich) 80 µg/mL in PBS with Ca^2+^/Mg^2+^, for 1 h.

After coating, 5 × 10^4^ cells in 5 µL of complete medium were seeded just on top of the recording electrodes and were allowed to attach. After 1 h, 400 µL of Complete Ncyte CNS Medium was gently added to each well. The medium exchange was performed every other day replacing 50% of the medium.

### 2.3. LEV Treatment, MEA Recordings, and Data Processing

The spontaneous firing was recorded every week for up to 6 weeks, and the experiments were performed when hCNS Neuron Cells showed a synchronous activity pattern network (at week 5 or 6).

LEV was dissolved in PBS to obtain a 100 mM (100×) solution; then, a hundredth of cell medium was taken off the MEA dishes and replaced with the same volume of LEV (final concertation: 1 mM); the addition of this amount of PBS to the culture medium does not affect the neuronal cells. Cell activity was monitored before and after the addition of 1 mM LEV with 2 min recording every 3 min, up to 20 min. After the last recording of the treatment phase, LEV was removed by replacing the cell medium with Complete Ncyte CNS Medium: after the washout, 5 recordings of 2 min were performed every 30 min and one at 24 h. During the recordings, cells were continuously at 37 °C under a 5% CO_2_ atmosphere. Four independent experiments were performed under the same experimental condition.

Neural activity was recorded and analyzed by Maestro Edge MEA (Axion Biosystems) equipped with Axion Integrated Studio (AxIs) Navigator 3.2.3.1 software (Axion Biosystems). Briefly, to record spontaneous neuronal activity we selected the Neural Real-Time module. The spiking activity was defined applying an Adaptive Threshold Crossing method, that sets the threshold for the spike detection to 6× Std Dev of the noise. This recommended threshold is optimal to minimize both false-positive and missed spike detections. The sampling frequency for spikes detection was 12.5 kHz.

To detect single-electrode bursting activity, an Inter-Spike Interval (ISI) Threshold was used, setting the minimum number of spikes at 5 and the maximum ISI at 100 ms. The network bursting activity was analyzed by the Neural Metric Tool (Axion Biosystems). For this purpose, the Envelope algorithm was selected using the following settings: Threshold Factor = 1.25; Min IBI = 100 ms; Min Electrodes = 35%; Burst Inclusion = 75%. When needed, settings were adjusted based on the actual precision of the detection. All metrics were exported as .csv and data were imported in GraphPad PRISM 9.2 to perform statistical analysis.

### 2.4. Analyzed Variables

Regarding spiking of neuronal cell culture, the investigated variables were the number of spikes and the coefficient of variation of the inter-spike interval (ISI coefficient of variation). The number of spikes refers to the total number of spikes recorded during the analysis. The ISI coefficient of variation is a measure of spike regularity since it expresses the coefficient of variation of the time interspersed between spikes.

Relative to single-electrode burst, number of bursts, burst duration, number of spikes per burst, mean ISI within burst, coefficient of variation of inter-burst interval (IBI coefficient of variation), and burst percentage were analyzed. Briefly, the number of bursts indicates the total number of single-electrode bursts over the duration of the entire recording window. The burst duration is the average time (expressed in seconds) from the first to the last spike in a single-electrode burst. The number of spikes per burst reflects the average number of spikes in a single-electrode burst. Mean ISI within burst describes the average time between spikes, for spikes within a single-electrode burst; the smaller the value, the more intense the burst. The IBI coefficient of variation is a measure of single-electrode burst regularity. Finally, burst percentage is the number of spikes in a single-electrode burst divided by the total number of spikes, multiplied by 100: so, burst percentage describes how many spikes form the bursts and, consequently, how many spikes do not.

Regarding network bursting behavior, the number of network bursts, number of spikes per network burst, and mean ISI within network burst were analyzed. A network burst represents a coordinated cluster of spiking across multiple electrodes. The number of network bursts describes how many network bursts are formed during the entire analysis. The number of spikes per network burst indicates the average number of spikes occurring within a network burst, and the mean ISI within a network burst reflects the average time interspersed between spikes in a network burst.

### 2.5. Statistical Analysis

The analysis of variance (ANOVA) was used to check statistical significance differences between the time points. The eta-squared (η^2^) was used to describe the effect size of the differences. Statistically significant results underwent a post-hoc Dunnett’s multiple comparison test to check where the difference occurred, compared to a baseline recording. Tukey post-hoc was used to evaluate the differences in spike amplitude. All data are presented as mean ± standard error of the mean (SEM). Results were considered statistically significant when *p* < 0.05. All statistical analyses were performed with GraphPad PRISM 9.2.

## 3. Results

To provide an in-depth report of the neuronal activity both during and after the treatment with LEV, we analyzed the spiking, which describes the number and regularity of action potentials occurrence; the single-electrode bursting, which defines the occurrence of bursts (i.e., a cluster of spikes); the network bursting which encompasses several variables related to the network bursts, which are coordinated clusters of spiking across multiple electrodes. A qualitative analysis of single-electrode bursting, network bursting activities, and the evaluation of multiple extracellular spikes amplitude were also performed.

### 3.1. Spiking

**Figure 2:**
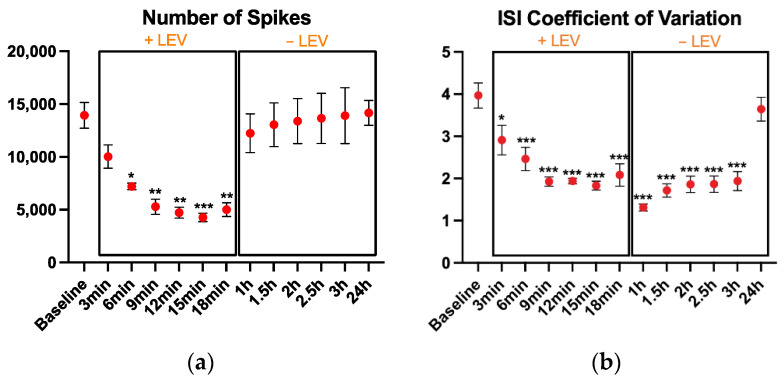
Spiking results: (**a**) the number of spikes, (**b**) coefficient of variation of the inter-spike interval during the 2 min recordings. After the baseline recording, LEV was added to the cell culture and its effects were monitored every 3 min up to 18 min; then, cell medium was replaced to remove LEV and cell activity was monitored every 30 min up to 3 h and finally, one recording was performed at 24 h. The cultures were kept at 37 °C under a 5% CO_2_ atmosphere during the recordings. Data are expressed as mean ± SEM of four independent experiments. All comparisons are performed vs. the baseline. * *p* < 0.05; ** *p* < 0.01; *** *p* < 0.001.

LEV addition to the cell culture-induced important modifications of the neuronal activity. We found that the number of spikes (Figure 2a) was different among the time points (*p* < 0.001, η^2^ = 0.69). Compared to the baseline, lower number of spikes were registered after 6 min (*p* = 0.03), 9 min (*p* = 0.003), 12 min (*p* = 0.001), with the lowest number of spikes recorded at 15 min (*p* < 0.001). The neural activity recovered after the LEV washout, and 40 min after the LEV removal, the number of spikes returned to baseline levels.

Also, the ISI coefficient of variation (Figure 2b) changed with the drug administration, showing a larger effect size (*p* < 0.001, η^2^ = 0.797). The ISI coefficient of variation was rapidly affected by LEV treatment, showing a significant reduction after only 3 min of treatment (*p* = 0.013) and remaining lower from then on. The recovery of the ISI coefficient of variation after LEV removal was very slow and only after 24 h, it returned to baseline values.

### 3.2. Single-Electrode Bursting

A single-electrode burst [14] was defined when at least 5 spikes occurred on an electrode, with each spike separated by an ISI of no more than 100 ms.

**Figure 3:**
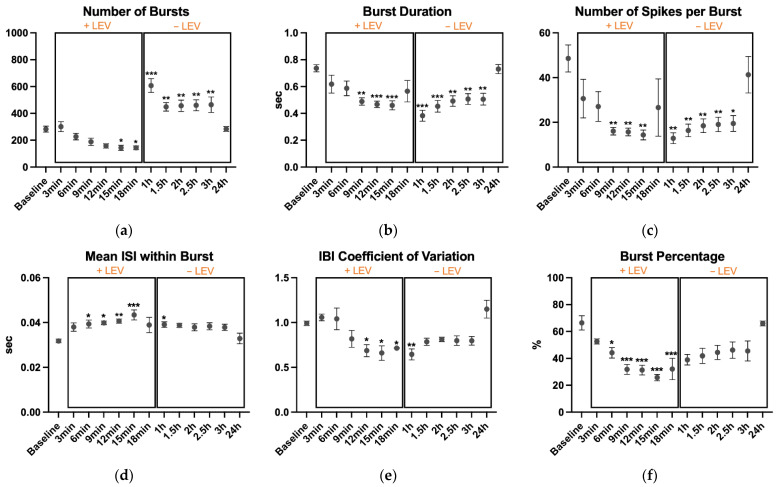
Single-electrode bursting results: (**a**) the number of bursts, (**b**) burst duration, (**c**) number of spikes per burst, (**d**) mean inter-spike interval within burst, (**e**) inter-burst interval coefficient of variation, and (**f**) burst percentage during the 2 min recordings. After the baseline recording, LEV was added to the cell culture and its effects were monitored every 3 min up to 18 min; then, cell medium was replaced to remove LEV and cell activity was monitored every 30 min up to 3 h and finally, one recording was performed at 24 h. The cultures were kept at 37 °C under a 5% CO_2_ atmosphere during the recordings. Data are expressed as mean ± SEM of four independent experiments. All comparisons are performed vs. the baseline. * *p* < 0.05; ** *p* < 0.01; *** *p* < 0.001.

The number of bursts (Figure 3a) was different among the time points (*p* < 0.001, η^2^ = 0.726). Compared to baseline, LEV lowered the number of bursts at 15 min (*p* = 0.04) and 18 min (*p* = 0.04). On the contrary, number of bursts were higher after replacing the medium, at 1 h (*p* < 0.001), 1.5 h (*p* = 0.008), 2 h (*p* = 0.005), 2.5 h (*p* = 0.004), and 3 h (*p* = 0.003). However, this sudden increasing in single-electrode bursting activity was likely due to the cell sensitivity to medium replacement, as seen in previous recordings (data not shown). Differences were also found for burst duration (*p* < 0.001, η^2^ = 0.418, Figure 3b), number of spikes per burst (*p* = 0.001, η^2^ = 0.521, Figure 3c), mean ISI within burst (*p* = 0.006, η^2^ = 0.471, Figure 3d), IBI coefficient of variation (*p* < 0.001, η^2^ = 0.654, Figure 3e), and burst percentage (*p* < 0.001, η^2^ = 0.669, Figure 3f).

Burst duration was lower at 9 min (*p* = 0.002), 12 min (*p* < 0.001), and 15 min (*p* < 0.001) during treatment with LEV. Also, after medium replacement, the duration was lower than baseline recording, although the values showed an increasing trend until 24 h when the value returned to baseline. 

Compared to baseline, also the number of spikes per burst showed lower values at 9 min (*p* = 0.003), 12 min (*p* = 0.003), and 15 min (*p* = 0.002) of treatment and at 1 h (*p* = 0.001), 1.5 h (*p* = 0.004), 2 h (*p* = 0.007), 2.5 h (*p* = 0.009) and 3 h (*p* = 0.01) after medium replacement.

Accordingly, the mean ISI within burst showed an increasing trend during the treatment. At 6 min (*p* = 0.038), 9 min (*p* = 0.022) and 12 min (*p* = 0.01), values were higher than baseline, and the higher value was detected at 15 min of treatment (*p* < 0.001). At 1 h, this metric was significantly higher (*p* = 0.047), while decreasing toward baseline levels from 1.5 h to 24 h.

On the other hand, the IBI coefficient of variation was significantly lower at 12 min (*p* = 0.021), 15 min (*p* = 0.01), and 18 min (*p* = 0.045) when cells were treated with LEV. At 1 h, the IBI coefficient of variation was still significantly lower (*p* = 0.007) while began to return close to baseline values from 1.5 h to 24 h.

Finally, burst percentage started to significantly decrease at 6 min (*p* = 0.023). The percentage continued to be statistically reduced at 9 min (*p* < 0.001), 12 min (*p* < 0.001), 15 min (*p* < 0.001) and 18 min (*p* < 0.001), with 15 min representing the lower value.

### 3.3. Network Bursting

**Figure 4:**
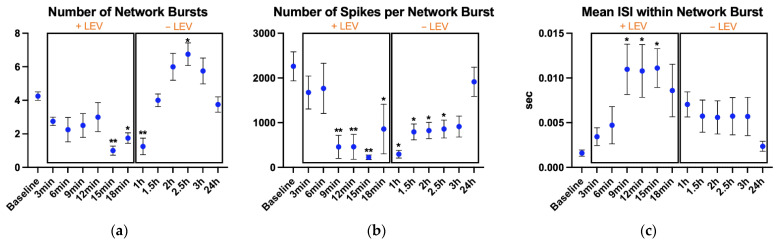
Network bursting parameters: (**a**) number of network bursts, (**b**) number of spikes per network burst, and (**c**) mean ISI within network burst. After the baseline recording, LEV was added to the cell culture and its effects were monitored every 3 min up to 18 min; then, the cell medium was replaced to remove LEV and cell activity was monitored every 30 min up to 3 h and finally, one recording was performed at 24 h. The cultures were kept at 37 °C under a 5% CO_2_ atmosphere during the recordings. Data are expressed as mean ± SEM of four independent experiments. All comparisons are performed vs. the baseline. * *p* < 0.05; ** *p* < 0.01.

Statistical analysis showed differences in the number of network bursts (*p* < 0.001, η^2^ = 0.57, Figure 4a), number of spikes per network burst (*p* < 0.001, η^2^ = 0.557, Figure 4b), and mean ISI within network burst (*p* = 0.025, η^2^ = 0.437, Figure 4c).

Differences for number of network bursts were found at 15 min (*p* = 0.003), 18 min (*p* = 0.043), and 1 h (*p* = 0.008), where they were lower than baseline recording. At 1.5 h, when the cells were in Complete Ncyte CNS Medium, the number of network bursts began to increase and the higher value was found at 2.5 h (*p* = 0.043), after which it started to decrease, and at 24 h, it returned comparable to baseline.

The number of spikes per network burst was considerably decreased by LEV compared to control. During the treatment, values were lower at 9 min (*p* = 0.004), 12 min (*p* = 0.004), 15 min (*p* = 0.002), and 18 min (*p* = 0.038). In addition, after medium replacement, the number of spikes per network burst were lower than baseline, at 1 h (*p* = 0.013), 1.5 h (*p* = 0.027), 2 h (*p* = 0.032), and 2.5 h (*p* = 0.038).

Finally, the mean ISI within network burst was higher than baseline during the treatment recordings, at 9 min (*p* = 0.022), 12 min (*p* = 0.025), and 15 min (*p* = 0.035).

### 3.4. Raster Plots: A Qualitative Description of Complex Neural Activity

**Figure 5:**
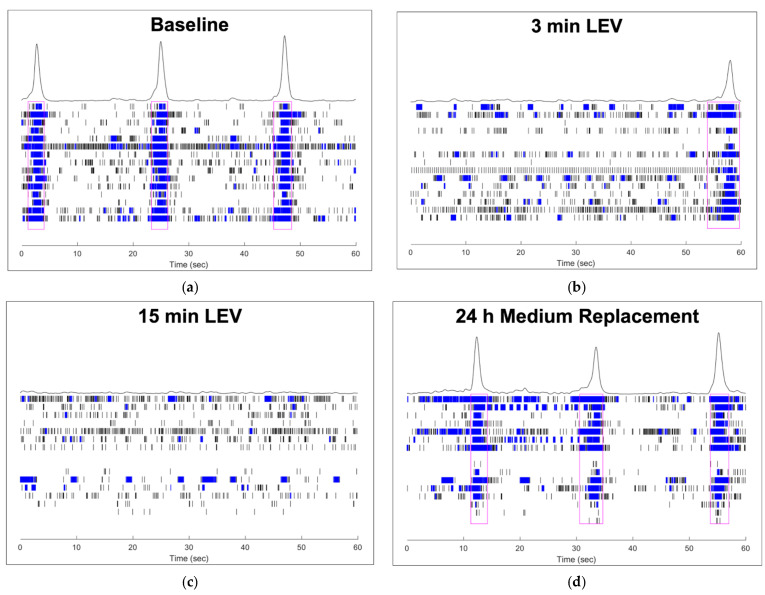
Raster plots: (**a**) baseline; (**b**) 3 min of treatment with LEV; (**c**) 15 min of treatment with LEV; (**d**) recording at 24 h with the basal medium. Black vertical lines indicate the spike occurrence, while blue rectangles represent the single-electrode bursts (clustered spiking activity), that can be of different duration based on how many spikes they contain. In (**a**,**b**,**d**), network bursts are highlighted by magenta rectangles surrounding the “column” of single-electrode bursts. Every lane of this column (representing the network) corresponds to single-electrode recordings (in this case we used a 24-Well CytoView MEA plate that has 16 electrodes per well). In addition, the peaks over the networks reflect the overall amplitude of network bursts.

Raster plots can be used to have a clear qualitative insight into the spontaneous neural activity recorded by Maestro Edge MEA. In Figure 5, each panel is descriptive of one well during different timepoints (a = baseline recording, b = 3 min treatment, c = 15 min treatment, d = 24 h recording with only basal medium) of our experiment, and every image represents 60 s of spontaneous neural activity. When bursting activity occurs on multiple electrodes, a network burst is formed.

The baseline recording (Figure 5a) shows 3 clear network bursts (surrounded by magenta rectangles), that are interspersed by about 15 s. Between the network bursts, different electrodes showed the recording of single spikes and several single-electrode bursts. At 3 min of treatment with LEV, (Figure 5b) both the number of network bursts and whole network bursts amplitude decreased. Indeed, only one network burst is detected into the well during the 60 s, and spike activity began to diminish (in the upper half of Figure 5b, both spiking and single-electrode bursting reduction are very clear).

At 15 min of treatment (Figure 5c), no network bursts appeared in the raster plot, and some electrodes did not record activity during the 60 s; only spikes and several sporadic single-electrode bursts are recorded. On the contrary, at 24 h recording (Figure 5d), when cells were in basal medium without the antiepileptic treatment, the network burst pattern was comparable to baseline. Indeed, in Figure 5d, 3 network bursts are present, and the peaks over the networks display an amplitude increase, similar to the baseline raster plot.

### 3.5. Spike Amplitude

**Figure 6:**
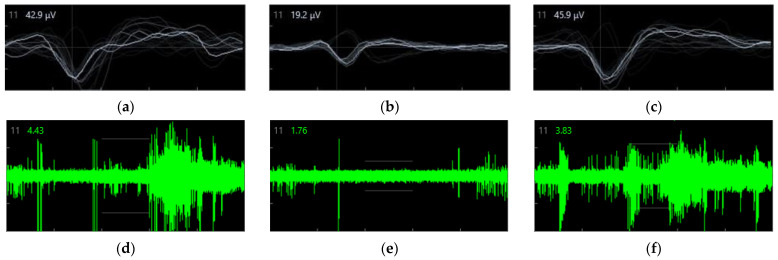
Spike amplitude recorded by the same electrode in three crucial different timepoints: in all three panels, the typical waveform of the EAP is evident. EAP waveform consists of three main distinct phases: (i) a brief, positive deflection; (ii) a larger negative peak; and (iii) a positive peak of longer duration. The first positive peak of the waveform is due to the positive capacitive current; the second, larger negative peak is the result of Na^+^ influx eliciting the internal action potential; and the last, longer positive peak mainly corresponds to the K^+^ efflux responsible for the cell repolarization. (**a**) baseline (**b**) after 15 min of treatment with LEV (**c**) at 24 h after medium replacement (on left upper angles in a, b, and c panels, light-grey numbers represent the electrode number, while white numbers correspond to the last EAP amplitude). Continuous waveform plot of the same electrode showing spiking activity at baseline (**d**), 15 min of treatment with 1 mM LEV (**e**), and at 24 h after medium replacement (**f**) (on left upper angles in d, e, and f panels, light-grey numbers represent the electrode number, and the green ones correspond the basal noise).

The spike amplitude (represented by the EAP waveform) data from the same well and electrode of the well, in three different crucial timepoints, were reported: baseline (Figure 6a), 15 min of treatment with LEV (Figure 6b), and 24 h after medium replacement (Figure 6c). These data represent a snapshot during the whole recordings. However, analyzing the data recorded by the same electrode provides meticulous information regarding the different electrophysiological activities. The AxIS Navigator allows one to export the raw data for a maximum of 20 spikes at any given time. Of note, these EAP phases can show slightly different shapes based on which part of the neuron the EAP is recorded [13,25], and also the distance from the electrode and the polar orientation of the cell could affect the waveform shape. To quantify the amplitude difference in the three crucial phases of the experiment, we calculated the mean peak amplitude (MPA) expressed in µV from the recorded spikes. We considered the MPA as the mean higher voltage recorded during the Na^+^ influx phase (i.e., large negative peak of the EAP). We would emphasize that usually, EAP amplitude is reported as an absolute value; however, here we present the data using negative values since the peak Na^+^ influx phase is represented by a negative deflection with respect to the beginning of the spike. Moreover, a qualitative representation of the LEV effect on spiking activity of the same electrode is shown in Figure 6 (d = baseline, e = 15 min LEV, f = 24 h after medium replacement).

Differences were detected between the three timepoints (*p* < 0.001, η^2^ = 0.601). At 15 min of treatment with LEV (Figure 6b), MPA was lower than the baseline (Figure 6a) (−13.11 ± 0.44 vs. −28.81 ± 1.96, *p* < 0.001) and the 24 h timepoint (Figure 6c) (−13.11 ± 0.44 vs. −26.87 ± 1.17, *p* < 0.001). No differences were found between baseline and 24 h.

## 4. Discussion

In our study, a comprehensive electrophysiological dataset of both the acute and delayed effects of LEV in human iPSC-derived neuronal cells containing a heterogeneous cell population (GABAergic, glutamatergic, dopaminergic, and astrocytes) was collected. This in vitro model is highly efficient since the presence of astrocytes allows the cell culture to reach a state of maturity that consents a good neurons functionality [10]. In addition, the synaptic communication of different neuronal populations makes this in vitro model more similar to an in vivo one. Indeed, spiking, bursting and network bursting activities detected in such a population with MEA, mimic the electrophysiological activity of in vivo models [21,22]. LEV has demonstrated multiple effects in different pathological conditions other than epilepsy [6,7]; thus, it is important to collect a further complex neural response to LEV in a nonclinical setting. In this regard, MEA offers an innovative way to detect important features of in vitro neuronal cell cultures other than spiking activity.

Our results demonstrate sharp modifications of complex electrophysiological metrics during several timepoints in hiPSC-derived neuronal cells in response to LEV treatment.

In recent years, the advancements in human stem cell technology allowed the in vitro generation of different cell type characterized electrical activity such as cardiomyocytes and neuronal cells [26,27,28]; in particular, by means of specific differentiation protocols various neuronal cell types have been derived from iPSC [29,30], and recently also from perinatal stem cells [31,32,33]. The electrical properties of these neuronal cells can be analyzed by MEAs, a technology that allows one to record spontaneous electrical activity for long time periods, in a non-invasive manner. The combined use of human stem cell-derived neurons and MEAs technology represents an optimal approach to test physiological and pathological neuronal cell activity, as well as drug effects and toxicity. In our study, we used a mixed population of human neurons (glutamatergic, GABAergic and dopaminergic neurons, and astrocytes) to obtain the most physiological environment to study the effect of LEV.

Our data evidenced that LEV led to a sharp decrease of spike number, and such decline was evident starting from 6 min of treatment, with the major decrease after 15 min. During the treatment, and until 3 h after medium replacement, the ISI coefficient of variation diminished and returned near baseline levels at 24 h. This means that LEV led to a decrease of spiking activity while regularizing the interval between spikes.

The bursting activity was also affected by LEV. The number of bursts showed a decreasing trend and was lower than baseline at 15 and 18 min of treatment. On the contrary, medium replacement elicited a two-fold increase of bursts. However, this finding is difficult to be interpreted: indeed, the electrical activity of neurons is highly affected in vitro by the medium change because of the cell sensitivity to modification of temperature, pH, and gaseous environment; this fact is particularly important in our case, as we had to substitute all the medium (and not only 50%) to remove the LEV. To avoid the bias related to these environmental factors, cells were allowed to re-equilibrate and the first recording to analyze the effect of the LEV washout was performed 30 min after the medium replacement. Nevertheless, it must be noted that 24 h after the LEV washout the number of bursts returned to initial levels. Burst duration also showed a decrease both during treatment and after medium replacement, although when LEV was eliminated, the burst duration began to raise. Such a decrease is the result of both diminished number of spikes and modification in the time between spikes elicited by LEV. Indeed, although the number of spikes was higher immediately after the medium replacement, the interval between spikes was more regular, resulting in the diminished time of spike clustering occurrence (i.e., bursting). This result is corroborated by the increased ISI within the burst during LEV treatment, showing a higher interval between spikes within the bursts. Also, the decreased IBI coefficient of variation during LEV treatment indicated an increased single-electrode burst regularity. Importantly, such responses reflect the effect of LEV in humans. Indeed, spike-wave burst numbers and frequency measured by video/electroencephalography showed a dramatic reduction after LEV treatment, while discontinuation causes a rapid increase [34].

The spike amplitude decreased during treatment, and at 15 min after LEV administration, peak amplitude showed a 50% reduction compared to baseline. Furthermore, spike amplitude returned to the baseline voltage the day after medium replacement. As previously reported, pharmacological treatment can modify EAP waveform and amplitude. Moreover, the EAP waveform varies depending on the different parts of the neurons (i.e., soma or neurites) and among the diverse neuronal cell types [16,17,18]. Accordingly, the fact that both EAP waveform and amplitude changed during LEV treatment could be due either to the modification of ionic channel behavior in response to the drug or the inhibition of certain neuron cell types (probably the most affected by the drug among the different cell typologies). Finally, MEAs detected that the network bursts were dramatically lower at 15 and 18 min of treatment and continue to show low values at 1 h. These data reflect a diminished synaptic communication between adjacent electrodes, which is confirmed qualitatively by raster plots (Figure 6). Previous investigations showed that LEV decreased synaptic transmission and that the effect of the antiepileptic was strongly mediated at the presynaptic level [35,36]. As expected, when network bursts were detected, MEAs results showed a lower number of spikes within the networks during LEV treatment, which remain low up to 2.5 h. This means that although the number of spikes increased, they occurred in an “intermittent” manner, probably due to a number of neurons still inhibited.

## 5. Conclusions

The present study provides a comprehensive overview of the electrophysiological response of a mixed population of human neurons to LEV administration. Exploiting a last-generation MEA system we were able to demonstrate in detail the inhibiting effect of the antiepileptic drug on both the neuronal spiking and the network connectivity. Our results confirm the high sensitivity of MEA technology in detecting drug-induced electrophysiological modifications. Moreover, these data allow one to extend the knowledge on the electrophysiological effects of LEV on the complex neuronal population that resembles the human cortex.

## Data Availability

Data are available on reasonable request from the authors.
